# Behavioral Evidence for Differences in Social and Non-Social Category Learning

**DOI:** 10.3389/fpsyg.2012.00291

**Published:** 2012-08-15

**Authors:** Lucile Gamond, Catherine Tallon-Baudry, Nicolas Guyon, Jean-Didier Lemaréchal, Laurent Hugueville, Nathalie George

**Affiliations:** ^1^CNRS, UMR7225Paris, France; ^2^Centre de Recherche de l’Institut du Cerveau et de la Moelle épinière, Université Pierre et Marie Curie-Paris 6, UMR-S975Paris, France; ^3^INSERM, U975Paris, France

**Keywords:** faces, first impressions, learning, person perception, social categorization

## Abstract

When meeting someone for the very first time one spontaneously categorizes the seen person on the basis of his/her appearance. Categorization is based on the association between some physical features and category labels that can be social (character trait…) or non-social (tall, thin). Surprisingly little is known about how such associations are formed, particularly in the social domain. Here, we aimed at testing whether social and non-social category learning may be dissociated. We presented subjects with a large number of faces that had to be rated according to social or non-social labels, and induced an association between a facial feature (inter-eye distance) and the category labels using two different procedures. In a first experiment, we used a feedback procedure to reinforce the association; behavioral measures revealed an association between the physical feature manipulated and abstract non-social categories, while no evidence for an association with social labels could be found. In a second experiment, we used passive exposure to the association between physical features and labels; we obtained behavioral evidence for learning of both social and non-social categories. These results support the view of the specificity of social category learning; they suggest that social categories are best acquired through unsupervised procedures that can be considered as a simplified proxy for group transmission.

## Introduction

Categorization is a fundamental skill of human cognition, which allows the organization of knowledge and therefore the appropriate processing of encountered items even when they are seen for the first time (Keri, 2003). Categorization is a pervasive process that seems to operate automatically, and even sometimes unconsciously, on objects as well as persons (Macrae and Bodenhausen, 2000). For instance, supposing that I did not know George Clooney before, I would still rapidly and effortlessly judge him as a white, tall, rather likeable looking man when meeting him for the very first time. Such first impressions are formed via knowledge activation which is derived from available visual traits and their existing associations with category labels, especially on a first encounter when no other information is available. Here we were interested in how such knowledge activation may take place when categorizing unknown faces.

The short example above reveals the wide range of categories that one may use to construe newly encountered persons. This range encompasses non-social categories (such as physical size) as well as social categories including personality traits such as likeability. Judging someone along social or non-social dimensions may rely on distinct neural networks. Medial frontal cortex areas seem to be specifically involved in social judgments: they are more activated when comparing persons along intelligence than when comparing them along size (Lindner et al., 2008) and are also more activated when subjects judged psychological characteristics as applicable to persons or dogs (pertaining to social semantics) as compared to situation where they judged body parts (pertaining to non-social semantics; Mitchell et al., 2005). Furthermore, anterior temporal lobes are more activated when judging the relation between social concepts than when judging the relation between vital function concepts (Zahn et al., 2007). Thus, overall, fMRI data emphasize the existence of a dedicated neuroanatomical network involved in the processing of social categories.

Social categorical judgments therefore seem to involve specific neural processes. Does the acquisition of the knowledge that underlies these judgments also obey specific rules? More specifically, do we learn to associate a physical feature with a social category in the same way that we learn to associate a physical feature with a non-social category? While a large number of studies have focused on how associations between visual features and non-social category labels are acquired (e.g., Posner and Keele, 1968; Salatas and Bourne, 1974; Ashby and Maddox, 1990; McKinley and Nosofsky, 1995; Freedman et al., 2001; Smith and Minda, 2001; Sigala and Logothetis, 2002; Nomura et al., 2007; for review see Schyns et al., 1998 and Ashby and Maddox, 2005), studies that have explored the learning of new social categories are much scarcer. These studies have particularly focused on the experimental manipulation of social categorical knowledge - they examined whether systematic, usually implicit, manipulation of the association between physical features and social category labels may bias the process of social categorization (Lewicki, 1986; Hendrickx et al., 1997; Barker and Andrade, 2006; Gamond et al., 2011). These studies have manipulated the association between a visual feature (e.g., hair length) and a personality trait (e.g., kindness). They have led to inconsistent behavioral results: Lewicki (1986) reported biased judgments with longer reaction times while Barker and Andrade (2006) observed faster reaction times to learnt categories. Another group of studies reported weak or negative behavioral findings (Hendrickx et al., 1997; Bos and Bonke, 1998; Gamond et al., 2011), although electrophysiological results recently revealed that the manipulated visual feature triggered specific neural processes, thereby indicating that it had been detected by the brain (Gamond et al., 2011). Thus, while many studies have shown evidence for perceptual category learning on objects, the few studies in the social domain have led to mitigated results. Potentially, this could indicate that social category learning relies on distinct processes as compared to those involved in non-social category learning. The aim of the present study was to compare directly the acquisition of an association between a visual feature and either a social or a non-social category label.

We investigated the extent to which subjects may learn to use a systematic association between a physical feature and a category label, contrasting social and non-social category labels, in two experiments with different association procedures. Subjects were presented with unknown, non-repeated faces that they had to categorize according to either a social category label or a non-social category label. The social category labels that we chose (“Flexible” and “Determined”) were of low-emotionality and similar in their affective valence. As for non-social category labels, we chose abstract labels (“A” and “B”) as non-social category labels. Such labels have been extensively used in previous studies of perceptual category learning (Salatas and Bourne, 1974; Homa and Cultice, 1984; Knowlton et al., 1996; Ashby et al., 2002; Maddox et al., 2008). Furthermore, we also chose to use “A” and “B” labels as they are not totally non-familiar: they are sometimes used in everyday life, notably for practical needs such as when distributing school kids into different classes or allocating university students to groups for laboratory practicals for example. We favored an “A” versus “B” judgment over a judgment based on a facial physical feature because these latter features are often associated with social characteristics: face shape is associated with competence judgments, or face width-to-height ratio to propensity to aggression (see, e.g., Zebrowitz and Montepare, 2008; Carré et al., 2009). A visual feature (inter-eye distance) was associated with either the social or the abstract category labels using two different association procedures. The first procedure was based on an explicit feedback given to the subject on a trial-by-trial basis (Experiment 1); this is a supervised learning procedure widely used in non-social visual category learning (Salatas and Bourne, 1974; Homa and Cultice, 1984; Knowlton et al., 1996; Ashby et al., 2002; Maddox et al., 2008). The second procedure (Experiment 2) was based on a passive exposure to the association akin to a simplified form of group transmission of information; such unsupervised procedure has been used previously for the study of social category learning (Lewicki, 1986; Barker and Andrade, 2006).

**Figure 1:**
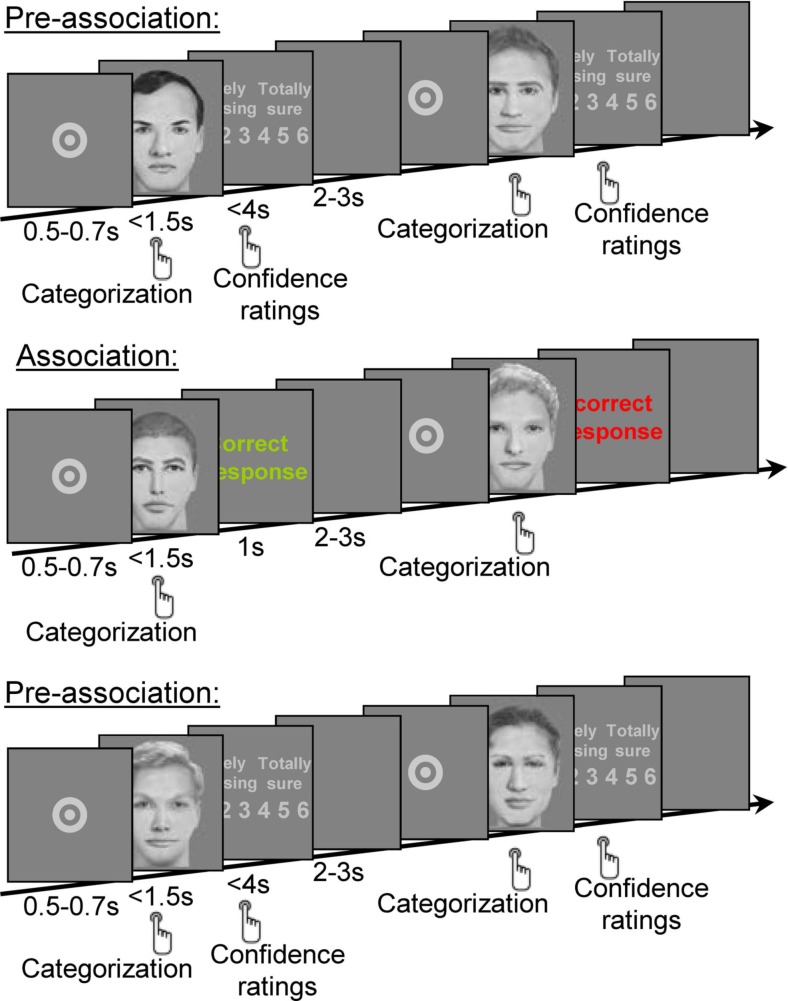
**Experiment 1, experimental design**. Each trial started with a central fixation point (presented for 0.5–0.7 s), followed by a face that subjects had to categorize as flexible or determined as quickly as possible and before the face disappearance. The face remained on-screen until subjects’ response or for up to 1.5 s. In both the pre- and post-association phases, a confidence rating screen was then presented. Subjects had to rate the confidence they had in their personality judgment between one (random guess) to six (absolutely sure). Inter-trial interval was initiated by subjects’ response or after up to 4 s. In the association phase, there was no confidence rating after the categorization response but subjects were given a 1 s feedback on the accuracy of this response. Unknown to the subject, the feedback was based on the inter-eye distance of the face presented. There was no stimulus repetition across the successive phases of the study; every face and every face feature (including the eyes) were new for each phase.

In each experiment, two different groups of subjects performed either the social or the non-social categorization task. Face stimuli (Gamond et al., 2011) consisted in two groups of faces, one with small and the other with large inter-eye distance, each face being unique and presented only once. Each experiment comprised three phases (Figure 1). During the whole procedure, subjects had to categorize faces and then rate their confidence in the judgment they gave. In the pre-association phase, that was used as a baseline control, the subjects performed the categorization task while being entirely naive with regard to the association between the physical feature (the inter-eye distance) and the category labels. In the association phase, the subjects were exposed to an arbitrary systematic association between the physical feature and the category labels. Finally, in the post-association phase, the subjects resumed the same categorization task as in the pre-association phase. We tested whether categorization performance in the post-association phase was modulated according to the reinforced association. Performance was examined in terms of accuracy, reaction times, and confidence ratings, the latter measure being potentially a more sensitive measure than accuracy (Persaud et al., 2007). We were mainly interested in the differences between correct and incorrect responses. We defined correct and incorrect responses according to the association rule to which the subjects were exposed in the association phase. Any difference between correct and incorrect responses in the post-association phase would indicate some degree of acquisition of the association between the category labels and the physical feature.

## Experiment 1

In this first experiment, the association phase was inspired from a previous study (Gamond et al., 2011) where the association between a physical feature, inter-eye distance, and two personality traits was reinforced via a feedback procedure. Feedback procedures have been extensively used in the study of perceptual, non-social, category learning (Salatas and Bourne, 1974; Homa and Cultice, 1984; Knowlton et al., 1996; Ashby et al., 2002; Maddox et al., 2008).

Under this procedure, during the association phase, subjects categorized each face and received an immediate feedback (“Correct response” or “Incorrect response”; Figure 1). Unknown to the subjects, this feedback was based on an association between the inter-eye distance (large versus small) and category labels (flexible versus determined in the social task, A versus B in the non-social task). This association phase therefore aimed at forming an association between the category labels and the inter-eye distance, as assessed by examining performance during the categorization of new faces along the chosen labels during the post-association phase.

Our aim in this experiment was to test for a differential influence of feedback depending on the social or non-social nature of the categorical judgment. Indeed, previous studies have indicated that feedback procedures are effective for non-social visual category learning (e.g., Knowlton et al., 1996; Maddox et al., 2008), whereas by contrast, our previous results (Gamond et al., 2011) showed no influence of feedback on social category learning (see also Bos and Bonke, 1998). Here, only social stimuli (faces) were used. Yet, in keeping with the above mentioned results, we expected that the feedback procedure may impact the performance in the non-social categorization task, while the social categorization task should remain unaffected by this procedure – or at least less affected than the non-social categorization task.

### Materials and methods

#### Participants

A total of 28 subjects participated in this experiment. Half of the subjects (eight females and six males in each group) underwent the social categorization task while the other half underwent the non-social categorization task. All subjects provided informed written consent and were paid 10€ per hour for their participation. Subject recruitment procedure was the same for the two groups to ensure that the subjects did not differ either in age [mean age for the social task subjects = 24.8 ± 1.1 years and for the non-social task subjects = 23.5 ± 0.8 years, unpaired *t*-test, *t*(26) = 0.96, *p* > 0.35] or in educational level [number of years of study after high-school = 3.9 ± 0.5 for the social task and =3.4 ± 0.4 for the non-social task; unpaired *t*-test, *t*(26) = 0.42, *p* > 0.4]. All participants but two were right-handed and all had normal or corrected-to normal vision.

#### Stimuli

**Figure 2:**
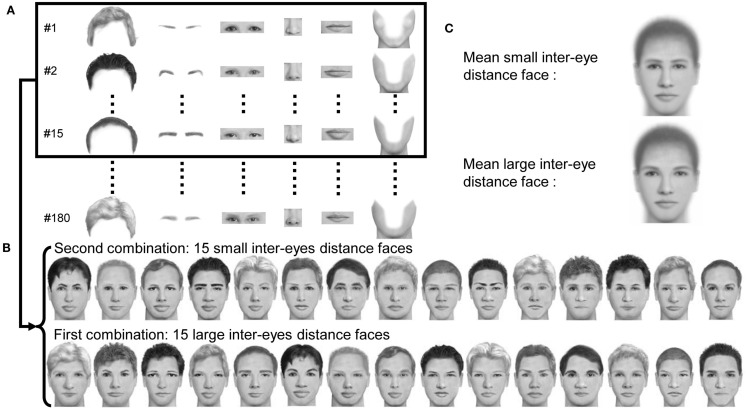
**Stimulus construction**. **(A)** Our face set was built out of a pool of 180 exemplars of different facial features. Each experimental block of 30 different faces was built from 15 exemplars of each feature. **(B)** We drew two combinations of these facial features with the sole constraint that across the two subsets of 15 faces, no face stimulus shared more than one facial feature with any of the other faces. In this example, within each column, faces shared the same eyes (and only this feature). Inter-eye distance was decreased in the first subset, and increased in the second subset. This procedure was repeated 12 times so that all the 180 features were used once. **(C)** Mean faces in the two conditions of inter-eye distance, showing that only this feature varied across the two conditions.

Three hundred and sixty face composites were created with FACES 4.0 software (IQ Biometrix; Gamond et al., 2011). As described in Figure 2, we selected 180 exemplars of each of the following face features: eyebrows, eyes, nose, mouth, and jaw as well as 30 haircuts repeated twice in three different colors. We then created 12 blocks of 30 faces out of the 180 exemplars of every feature. To build the 30 faces of a block, we first randomly selected 15 exemplars of each feature. By combining the facial features differently, two sets of faces were created, with the sole constraint that no face of the second set shared more than one feature with any face of the first set. Finally, the eyes of the first set of faces were moved away from each other resulting in the large inter-eye distance face pool (mean distance between the eyes = 1.41° ± 0.15° of visual angle). The eyes of the second set of faces were moved closer to each other to create the small inter-eye distance face pool (mean distance between the eyes = 1.21° ± 0.15° of visual angle). With this procedure for each block, we obtained two sets of faces made of exactly the same facial features, yet consisting in unique combinations of these features so that every face was unique. Furthermore, there was no low-level difference between the large and the small inter-eye distance face sets except the inter-eye distance *per se*. The procedure was repeated 12 times to create the 12 blocks of 30 faces. Thus, every block contained different faces and they were seen once at most across all phases of the experiment. The faces were presented on a gray background (luminance: 44.5 cd/m^2^). They covered a visual angle of 5° vertically and 3.6° horizontally.

#### Procedure

##### Social task

Participants were comfortably seated in front of a screen placed at 85 cm from their eyes. The experiment was divided into three phases, pre-association, association, and post-association phases. In each phase, participants had to categorize the presented faces as either flexible or determined. A definition of these personality traits was provided beforehand, ensuring that for both traits, the descriptions contained similar amount of positively and negatively connoted terms, with both the pros and cons of flexible and determined personality.

The task was the same throughout the pre- and post-association phase. In each trial, after a variable central fixation period of 0.5–0.7 s, a face stimulus was presented for 1.5 s maximum. The participant had to indicate, with a mouse, whether the face looked flexible or determined as soon as possible and before the face disappeared. Participants then had 4 s maximum to rate their confidence in this judgment on a scale from 1 (“purely guessing”) to 6 (“totally sure”), using a keypad. The inter-trial interval (blank screen) varied randomly between 2 and 3 s.

During the association phase, participants received a 1-s feedback immediately after each of their personality judgment which indicated “correct response” (in green) or “incorrect response” (in red). Subjects did not have to rate their confidence during this phase. The feedback corresponded to an arbitrary association between the large or small inter-eye distance and the flexible or determined personality trait respectively. The association was constant for a given subject and counterbalanced across subjects so that for half of the participants, correct responses associated small inter-eye distance with “flexible” response and large inter-eye distance with “determined” response; the association was reversed for the other half. Half of the subjects responded “flexible” with their index finger and “determined” with their middle finger; this stimulus-response mapping pattern was reversed for the other half of the participants, and it was orthogonal to the association between inter-eye distance and personality label.

The pre- and post-association phases comprised two blocks and the association phase included four blocks, for a total of eight blocks per subject. The blocks that composed the pre-association, association, and post-association phases were counterbalanced across subjects so that the 12 blocks were seen across subjects, in every phase. Within each block, the order of face presentation was randomized, so that a given facial trait was repeated (in a different face – see above) with a minimum of three intervening stimuli. As mentioned above, there was neither face nor face feature repetition across blocks.

At the end of the recording session, the participants went through a questionnaire. They were asked to rank five main face features (eyebrows, eyes, nose, mouth, global face shape) from the least to the most important for their flexible/determined judgment. Subjects were then asked to indicate which particular property was relevant for the two top-most important features that they had chosen (The proposed choices were: -for eyebrows: length, inter-eyebrows distance, thickness, shape; -for eyes: gaze, color, inter-eye distance, size; -for nose; size, position, shape, nostril visibility; -for mouth: length, thickness, position, difference between lips, and -for face shape: size, thickness of neck, height of forehead, shape of jaws). The aim of this questionnaire was to determine to what extent subjects were explicitly using information about inter-eye distance during the experimental task.

##### Non-social task

The procedure was exactly the same as in the social task except that personality categorization was replaced by a non-social categorization: the subjects had to indicate if each face belonged to category “A” or category “B.”

#### Data analysis

We analyzed the number of correct responses, as well as the median reaction times and mean expressed confidence levels for the correct and incorrect responses in each experimental condition. Correct responses were defined as responses that corresponded to the association introduced during the association phase.

First, for each subject, each type of response (Flexible or Determined in the social task; A or B in the non-social task), and in each phase, we excluded the trials in which RTs either exceeded or were below two standard deviations of the mean RT or with no response (a mean of 5.8 ± 1.6% of trials were excluded on this basis, across subjects)^1^. Then, we calculated the mean number of correct responses, the mean confidence levels of correct and incorrect responses, and the median RT of correct and incorrect responses, for every subject, in the post-association phase and – as a control – in the pre-association phase.

We analyzed accuracy by performing an unpaired *t*-test between tasks to compare the number of correct responses in the post-association phase of the social and non-social tasks. For each task, we also compared the mean number of correct responses to chance level (50% of the number of given responses) using a binomial test. We analyzed median reaction times and mean confidences ratings by performing an ANOVA with task as a between-subject factor and the type of responses (correct/incorrect) as a within subject factor. When significant effects were found we performed planned comparisons in each group of subjects (social and non-social task) according to our working hypothesis and controlled that the effect did not pre-exist to the association phase.

### Results

Accuracy, reaction times, and confidence ratings in the two tasks are presented in Table 1.

**Table 1:** **Results of experiment 1 and 2, for (a) post-association phase and (b) pre-association phase**.

		Nb of correct responses	Confidence ratings		Reaction times (ms)	
**A**						
Experiment 1: feedback procedure	Social	28.5 ± 1.4 (50.60%)	IR	3.92 ± 0.15	IR	898 ± 39
			CR	3.81 ± 0.15	CR	862 ± 30
	Non-social	27.9 ± 1.6 (54.89%)	**IR**	**3.68 ± 0.25**	IR	944 ± 37
			**CR**	**4.02 ± 0.18**	CR	915 ± 31
Experiment 2: passive procedure	Social	27.1 ± 1.1 (50.08%)	IR	4.20 ± 0.12	**IR**	**861 ± 26**
			CR	4.24 ± 0.12	**CR**	**836 ± 26**
	Non-social	27 ± 2.8 (52.89%)	IR	4.21 ± 0.22	**IR**	**807 ± 33**
			CR	4.21 ± 0.27	**CR**	**751 ± 48**
**B**						
Experiment 1: feedback procedure	Social	29.5 ± 0.9 (52.68%)	IR	3.98 ± 0.12	IR	876 ± 40
			CR	3.97 ± 0.14	CR	911 ± 33
	Non-social	25.0 ± 1.2 (48.28%)	IR	4.15 ± 0.21	IR	894 ± 36
			CR	4.29 ± 0.20	CR	883 ± 35
Experiment 2: passive procedure	Social	24.1 ± 1.3 (52.09%)	IR	4.19 ± 0.17	IR	802 ± 35
			CR	4.28 ± 0.12	CR	806 ± 36
	Non-social	23.8 ± 2.1 (47.30%)	IR	4.36 ± 0.15	IR	859 ± 28
			CR	4.39 ± 0.20	CR	840 ± 28

*Mean and standard error of the mean of number of correct responses (percentage of correct responses in brackets), confidence ratings and reaction times. IR, incorrect responses; CR, correct responses. Significant results are indicated in bold font*.

As detailed below, while accuracy and RTs did not vary, significant between tasks differences were observed on confidence ratings.

Accuracy was not significantly different between the social task and the non-social task (non-paired *t*-test: *t* = 1.16, *p* > 0.20). Moreover, the mean number of correct responses was not significantly different from chance level neither in social (mean number of correct responses = 28.5 ± 1.4 that is 50.60% of responses; binomial test, *z* = −0.04, *p* > 0.40) nor in non-social task (mean number of correct responses = 27.9 ± 1.6 that is 54.89 ± 2.91% of responses; binomial test, *z* = 0.56, *p* > 0.20).

Similarly, the statistical analysis of RT did not reveal any main difference between social and non-social tasks [*F*(1,26) = 1.18, *p* > 0.20], nor between correct and incorrect RTs [*F*(1,26) = 2.85, *p* > 0.10]. Interaction between these two factors was also non-significant [*F*(1,26) = 0.03, *p* > 0.50].

**Figure 3:**
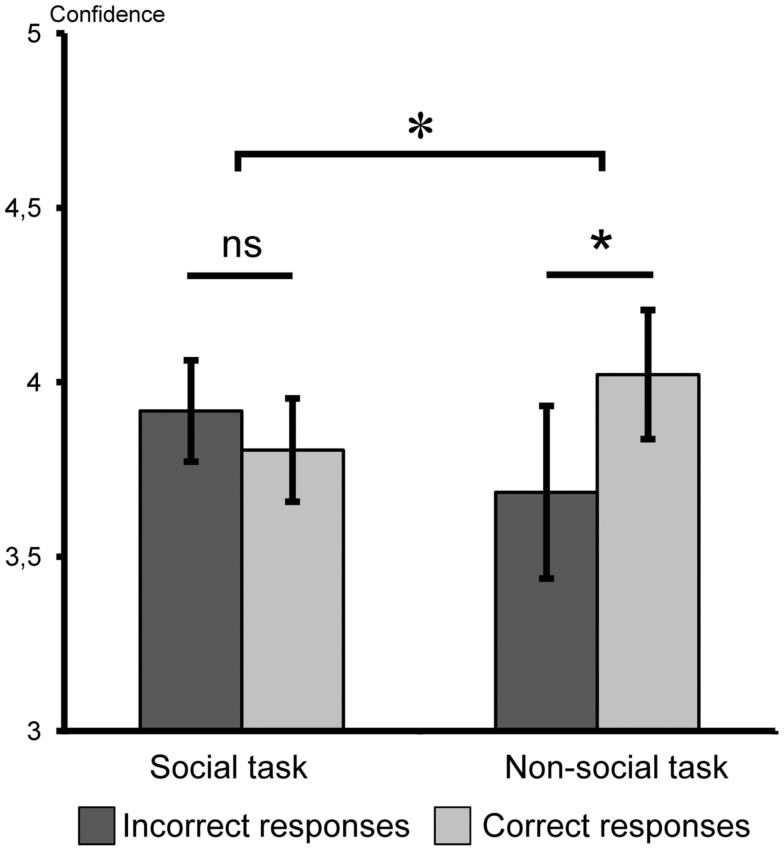
**Experiment 1, mean confidence ratings for correct and incorrect responses in the post-association phase for social and non-social categorization tasks**. In light gray, correct responses; in dark gray, incorrect responses; *p < 0.05; ns, non-significant. Error bars indicate standard error of the mean.

However, the analysis of confidence ratings revealed an influence of the association on confidence judgments that was different in the social and non-social tasks. We did not observe any main effect of task [*F*(1,26) = 0.001, *p* > 0.50] and type of responses [correct/incorrect responses, *F*(1,26) = 1.50, *p* > 0.20], but there was a significant interaction between type of task and confidence ratings of correct versus incorrect responses in post-association phase [*F*(1,26) = 5.96, *p *< *0.03*; Figure 3]. Planned comparisons revealed that the influence of the association on confidence judgments was selective to the non-social task. Indeed, in the social task, subjects’ confidence in their personality judgments was not significantly different between correct and incorrect responses [mean confidence for correct responses = 3.81 ± 0.15 and for incorrect responses = 3.92 ± 0.15; paired *t*-test: *t*(13) = −1.05, *p* > 0.30], whereas in the non-social task, subjects’ confidence in their personality judgment was modulated by the association phase: confidence ratings were significantly higher for correct responses (4.02 ± 0.19) than for incorrect responses [3.68 ± 0.25; paired *t*-test: *t*(13) = 2.24, *p* < 0.05; Figure 3]. This result shows that the social and non-social tasks differed in their sensitivity to the feedback association procedure.

In order to confirm that these effects were induced by the association phase and could not be attributed to some differences pre-existing to our experimental manipulation, we performed the same analysis procedure on the pre-association phase. This analysis did not reveal any significant main effect [Task: *F*(1,26) = 1.13, *p* > 0.20; Type of response: *F*(1,26) = 0.73, *p* > 0.40] nor significant interaction [*F*(1,26) = 0.87, *p* > 0.30].

As effects on confidence ratings are usually observed in parallel with accuracy improvement, we checked if these two measures were correlated. We computed the correlation between percentages of correct responses and difference in confidence ratings for correct versus incorrect responses during the post-learning phase. This analysis revealed that, during this phase, the higher the accuracy, the larger the effect on confidence ratings was (Pearson correlation: *r* = 0.63 *p* < 0.02). In other words, those subjects who answered more often correctly (a clear indication of learning) also showed the largest confidence effect. This result supports the view that confidence ratings may be a more sensitive measure than categorical responses to reveal learning effects.

The post-experimental questionnaire revealed that a large majority of subjects had paid attention predominantly to the eye region in both tasks. However a more detailed analysis revealed that subjects behaved quite differently in the two tasks. In the social task all subjects had paid attention to the eye regions, as the majority of them (10 subjects out of 14) reported paying attention either to gaze direction or to eyebrows shape. One of the subjects reported using the inter-eye distance for performing the categorization task. By contrast, in the non-social task a smaller number of subjects (9 of 14 subjects) reported paying attention to the eye regions and among them four said they had used the inter-eye distance to perform the task. In order to test the possibility of a contribution of the conscious detection of the association rules to our measures of task performance, we assessed the influence of these subjects’ performances on the observed effects. First, accuracy level did not seem to be related with the report of using inter-eye distance: Two subjects out of the four subjects who reported using this feature reached an accuracy level of more than 60%. The two other subjects who mentioned this feature did not show accuracy above chance level. Moreover, we noted that two other subjects who did not report using inter-eye distance showed above 60% accuracy. Second, in order to test if the effect observed on confidence ratings was driven by the subjects who reported using inter-eye distance, we reproduced our statistical analysis excluding these subjects. We observed that the key interaction between task and correctness of response was maintained [*F*(1,22) = 5.22, *p* < 0.05] in the subjects who did not mentioned inter-eye distance in the post-experiment questionnaire (10 subjects in the non-social task and 14 subjects in the social task). The planned comparison of confidence ratings for correct versus incorrect responses in the non-social task only approached significance [*t*(9) = 2.11, *p* = 0.06] probably due to the reduced statistical power under this condition. Thus it did not seem that the subjects who reported using inter-eye distance drove the effect observed on confidence ratings.

### Interim discussion

Our aim was to examine category acquisition using a feedback procedure. There was no influence of feedback procedure on either reaction times or accuracy, but we found a difference between confidence ratings of correct and incorrect answers, thus demonstrating a form of acquisition of the reinforced association. Most importantly, this effect was dependent on the nature of the category labels employed: confidence judgments were affected only in the non-social task. This reveals an impact of the association phase on categorical knowledge in the non-social task, while no evidence of such an impact could be observed in the social task.

This experiment shows that feedback association procedure can be efficient to alter categorical judgment on social stimuli such as faces. The reason why the measure of confidence ratings is the sole measure to reveal this dissociation remains unclear but the correlation that we observed between accuracy and confidence ratings is in line with the view that confidence ratings can be a more sensitive measure than the accuracy of categorical responses (Persaud et al., 2007). More importantly, the results point toward a dissociation between social and non-social categories: reinforcing by feedback an association between a physical feature and a category label did induce a detectable behavioral bias in the non-social task but not in the social task. The explanation for this dissociation between the social and non-social categorization tasks may be twofold.

First, social categories might be more resistant to learning. This interpretation is consistent with the comparison of category learning studies in the general perceptual field and in the social field. Studies in general perceptual domain have widely demonstrated that new visual categories of objects can be created in experimental settings (e.g., Posner and Keele, 1968; Ashby and Maddox, 1990; Shin and Nosofsky, 1992; Smith and Minda, 2001; for review see Ashby and Maddox, 2005). By contrast social psychology studies yielded mixed results regarding the learning of laboratory-manipulated social categories (Lewicki, 1986; Hendrickx et al., 1997; Barker and Andrade, 2006; Gamond et al., 2011). This relative resistance of social categories to learning could be linked to the existence of prior social knowledge that might prevent or interfere with the learning of new social categories. Indeed, Tajfel (1982) has suggested that a characteristic of social judgments may lie in the existence of a large body of knowledge on which these judgments are based. In other terms, learning to associate a physical feature with a personality trait may be difficult because any personality trait is already associated to a wealth of perceptual information. We checked that the subjects did not have pre-existing shared stereotypes linking inter-eye distance and flexible/determined personality traits. However this does not rule out prior idiosyncratic knowledge linking these personality traits to other physical characteristics. Note that this assumption is in agreement with the post-experimental questionnaire results in which a majority of subjects reported shape of eyebrows and gaze direction as important for the social categorization task. This might suggest the existence of prior knowledge linking eyebrows and gaze direction to the personality traits that we used.

Secondly, some authors have proposed that learning mechanisms are directly related to the nature of the acquired categories (Ashby and Maddox, 2005; Cunningham and Zelazo, 2007). This would then suggest that the feedback procedure that we used was more suited to the non-social than social category learning. In other words, feedback procedure may not be suitable for social category learning. In line with this view, a previous neuroimaging study of our group using the same paradigm did not show any effect on response times or accuracy (Gamond et al., 2011). Furthermore, another study that attempted to create an association between a physical feature and a social trait using feedback obtained a weak and transient behavioral effect of experimental manipulation (Bos and Bonke, 1998). In this latter study, participants were asked to intuitively judge the intelligence associated with first names. After each judgment, participants received feedback on the accuracy of their response. Unknown to the participants, this feedback was based on the presence of the letter “a” or “i” in the first name. A learning effect was observed in only one out of four blocks of the experiment. Moreover some studies have suggested that category learning through a feedback procedure relies on neural mechanisms that notably involve basal ganglia (e.g., Shohamy et al., 2004; Smith and McDowall, 2006; Seger and Miller, 2010). This region has been linked to automatic components of feedback-based learning during non-social categorization tasks (Satpute and Lieberman, 2006; Smith and McDowall, 2006). Furthermore, in the case of social categories, it seems that this region might only be involved for very few categories, which are automatically activated during person perception (Satpute and Lieberman, 2006). Such automatic category activation would concern only the categories of gender, age, and ethnicity (Brewer and Lui, 1989). Thus, overall, feedback learning might not be suited for the learning of most social categories, including the flexible/determined categories used here.

In summary, our results showed a dissociation between social and non-social category learning using a feedback procedure. The results indicated an influence of the feedback on non-social categorization, while social judgments remained unaffected. This pattern of results suggests a specificity of social category learning. However, it relies on a null result in the social domain that might be explained by a general resistance of social categories to learning. Alternatively, social category learning may arise under other association procedures. Experiment 2 aimed at testing this hypothesis.

## Experiment 2

Following the hypothesis that learning mechanisms may be directly related to the nature of the acquired categories (Ashby and Maddox, 2005; Cunningham and Zelazo, 2007), we tried to devise an association procedure that may yield observable behavioral effects in the social categorization task. Social psychology literature emphasizes two properties of social category learning which may be interesting to transpose experimentally.

First, under natural conditions, we seldom get immediate feedback on social judgments. It rather seems that we are exposed to associations in the environment without explicitly seeking to identify rules and repetitions. Such passive exposure to an association could be translated in terms of experimental methodology into a non-supervised passive learning paradigm, Interestingly, the only studies that revealed a bias on categorical judgments accuracy after experimental manipulation of social association have used a passive learning procedure (Lewicki, 1986; Barker and Andrade, 2006). More specifically, these studies presented faces associated with short behavior descriptions that allowed inferring a personality trait. Subjects had simply to listen attentively to these descriptions while they fixated the faces.

Second, social psychology studies have suggested that social knowledge may be transmitted by the group to which we belong: we would be influenced by our peers. For instance, stereotypes may be transmitted by recurrent behaviors and judgments of the in-group regarding out-groups (Bandura et al., 1963). A simple implementation of this consists in indicating to subjects how the face has previously been categorized by a representative panel of judges.

We therefore tested whether a passive exposure to the association between a physical feature and a category label together with a very simplified form of group transmission of information could influence social categorical judgments.

**Figure 4:**
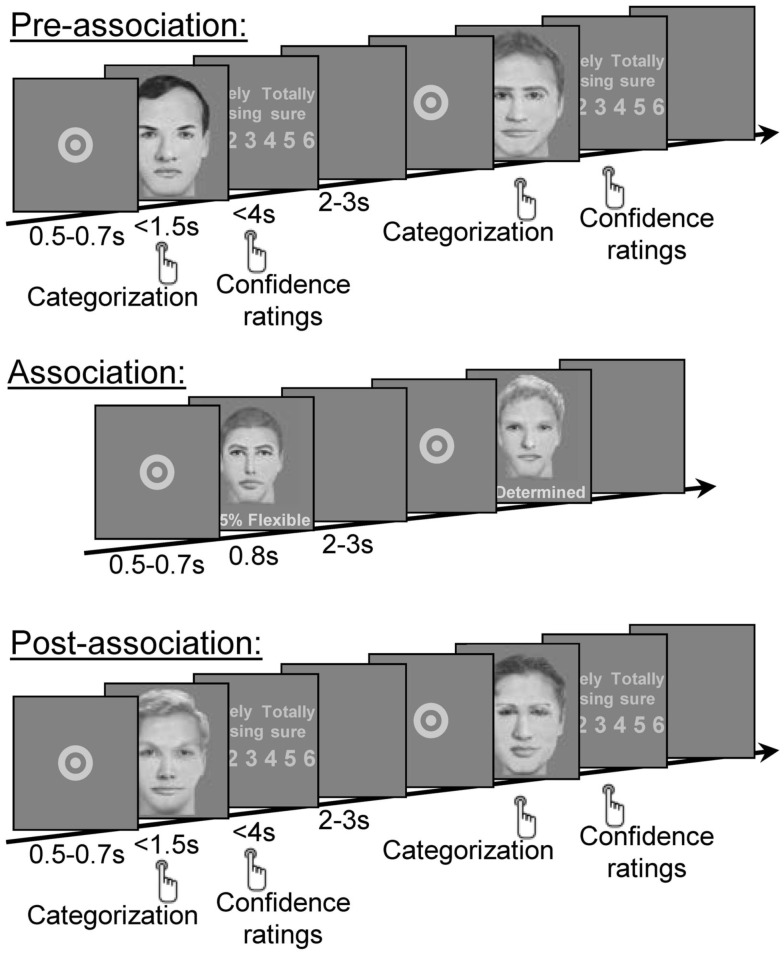
**Experiment 2, Experimental design of social categorization task**. In pre- and post-association phase, each trial started with a central fixation point, followed by a face that subjects had to categorize as flexible or determined as quickly as possible and before the face disappearance. The face remained on-screen until subjects’ response or for up to 1.5 s. In pre- and post-association phase, a confidence rating screen was presented. Subjects had to rate the confidence they had in their personality judgment between one (random guess) to six (absolutely sure). Inter-trial was initiated by subjects’ response or after up to 4 s. In the association phase, after the fixation point a face was presented with the percentage of persons that had categorized this face as flexible or determined. Unknown to the subject, narrow inter-eye distance faces were systematically paired with a high percentage for one personality trait, while large inter-eye distance faces were systematically paired with a high percentage for the other personality trait, to induce an arbitrary association between personality trait and physical feature. There was no stimulus repetition across the successive phases of the study; every face and every face feature (including the eyes) were new for each phase. This procedure was replicated identically for non-social categorization task (with the category labels A and B).

To test this hypothesis, we replicated the experimental protocol of Experiment 1 but modified the way subjects were exposed to the association (Figure 4). In the social categorization task, after completing the pre-association phase, subjects were informed that the persons whose faces were presented had been previously categorized by another panel of subjects as either flexible or determined persons. Then, in each trial of the passive exposure phase, a face was presented along with a personality trait and a percentage representing the proportion of subjects who previously categorized that face as having this personality trait. The percentage was randomly chosen between 75 and 90%. This procedure aimed at enforcing a simplified form of group transmission. As in experiment 1, unknown to the subjects, we introduced a systematic association between the physical feature of inter-eye distance (small or large) and the personality traits (flexible or determined). The pre- and post-association phases were strictly identical to pre- and post-association phases of experiment 1.

A non-social version of the task was performed by a different group of subjects. The protocol was identical except that subjects had to categorize the faces as belonging to category “A” or “B,” which were described as two abstract, arbitrary categories.

### Materials and methods

#### Participants

A total of 28 subjects participated in this experiment. Half of the subjects (eight females and six males in each group) underwent the social categorization task while the other half underwent the non-social categorization task. All subjects gave a written consent and were paid 10€ per hour. The same procedure of subject recruitment was used for the two tasks in order to ensure that the subjects from the two groups did not differ either in age [mean = 25.4 ± 1.2 years for the social task group and 23.4 ± 0.6 years for the non-social task group; unpaired *t*-test, *t*(26) = 1.60, *p* > 0.13] or in educational level [number of years study after high-school = 4.0 ± 0.5 for the social task subjects and = 3.7 ± 0.4 for the non-social task subjects; unpaired *t*-test, *t*(26) = 0.65, *p* > 0.40]. All participants were right-handed and had normal or corrected-to normal vision.

#### Stimuli

We used the same stimuli as in experiment 1.

#### Procedure

##### Social task

The procedure was similar to that of experiment 1 except with respect to the association phase which consisted in a passive exposure to the association between the physical feature and the personality trait.

The pre-and post-association phases were strictly identical to pre-and post- association phases of experiment 1 respectively. During the association phase (120 trials, ∼8 min), we explained to the subject that the faces he/she would now see had already been categorized by another group of participants as flexible or determined and that this information would be displayed to him/her simultaneously with each face. In each trial, after a fixation point presented for a variable period from 0.5 to 0.7 s, a face was presented (during 0.8 s) along with a percentage and a personality trait (flexible or determined) representing the percentage of persons who had previously categorized the face as flexible or determined (e.g., “85% flexible”); this information was displayed right underneath the face. The subject had no response to provide; he/she was instructed to carefully fixate the faces and the associated information. The inter-trial interval (blank screen) varied randomly between 2 and 3 s.

Unknown to the subject, the concomitant presentation of personality trait, percentage, and face was artificially constrained so that it introduced an arbitrary association between the personality trait and the physical feature of inter-eye distance. In half of the subjects, small inter-eye distance faces were presented with comments such as “80% flexible” and large inter-eye distance faces with comments such as “90% determined;” for the other half of the subjects, the association between the physical feature and the personality trait was reversed. The percentages were randomly chosen between 75 and 90%.

##### Non-social task

The procedure was strictly identical to the social task except that personality labels were replaced by non-social, abstract labels, “A” or “B.”

#### Data analysis

Data analysis was identical to that in experiment 1.

### Results

Accuracy, reaction times, and confidence ratings in the two tasks are presented in Table 1.

As detailed below, while accuracy and confidence ratings did not vary, significant effects were observed on RTs.

Accuracy was not significantly different between the social task and the non-social task [non-paired *t*-test: *t* = 0.60, *p* > 0.5]. Moreover, the mean number of correct responses was not significantly different from chance level neither in social (mean number of correct responses = 27.1 ± 1.1 or 50.08%, binomial test, *z* = −0.13, *p* > 0.40) nor in non-social task (mean number of correct responses = 27.0 ± 2.8 or 52.89%, binomial test, *z* = 0.36, *p* > 0.2).

Similarly, the association phase did not influence confidence ratings. Data analysis did not reveal any main difference between social and non-social task [*F*(1,26) = 0.00, *p* > 0.90] nor between correct and incorrect responses [*F*(1,26) = 0.05, *p* > 0.80]. Interaction between these two factors was also non-significant [*F*(1,26) = 0.03, *p* > 0.80].

**Figure 5:**
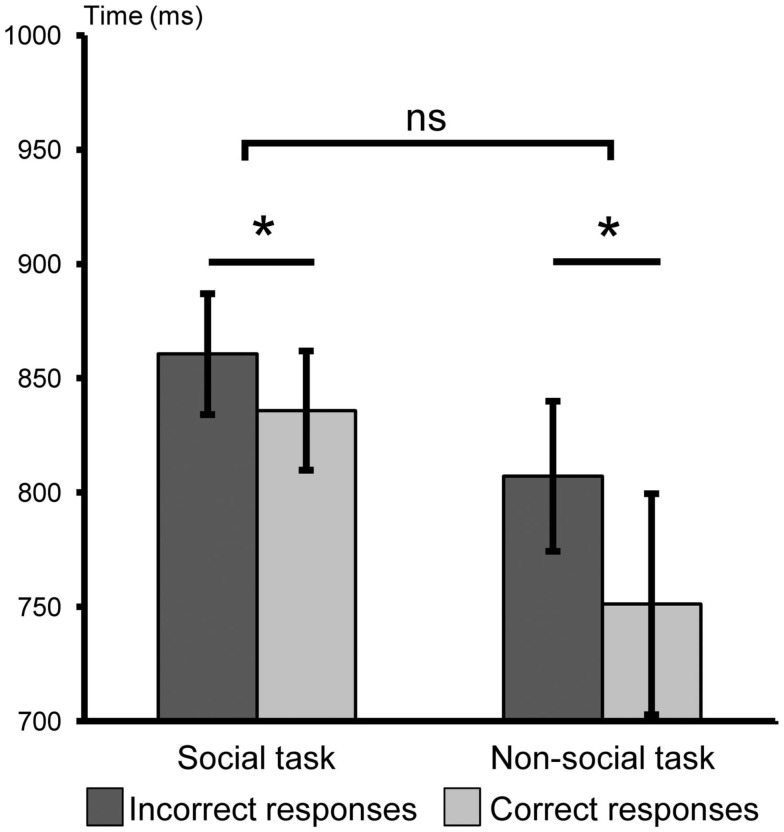
**Experiment 2, mean reaction times of correct and incorrect responses in the post-association phase for social and non-social categorization tasks**. In light gray, correct responses; in dark gray, incorrect responses; *p < 0.05; ns, non-significant. Error bars indicate standard error of the mean.

However, reaction times were altered by the association phase. RT analysis did not reveal main effect of task [*F*(1,26) = 2.13, *p* > 0.15] but revealed a significant main effect of response type: reaction times were significantly shorter for correct than for incorrect responses [*F*(1,26) = 10.03, *p* < 0.005; Figure 5] without any interaction between this independent variable and the type of task [social or non-social; *F*(1,26) = 1.51, *p* > 0.20]. Planned comparisons confirmed that RTs were significantly altered by the association phase in both tasks. We found that RTs were significantly shorter for correct than for incorrect responses in both the social [correct responses: 836 ± 26 ms; incorrect responses: 861 ± 26 ms; paired *t*-test: *t*(13) = 2.52, *p *< 0.03] and the non-social tasks [correct: 751 ± 48 ms; incorrect responses: 807 ± 33 ms; paired *t*-test: *t*(13) = 2.38, *p *< 0.04].

To ensure that these effects were induced by the association procedure we checked that they were absent in the pre-association phase. This analysis did not reveal any significant main effect [Task: *F*(1,26) = 1.18, *p* > 0.20; Type of response: *F*(1,26) = 2.21, *p* > 0.60] or significant interaction [*F*(1,26) = 0.49, *p* > 0.40]. The difference of reaction times for correct and incorrect responses observed both tasks thus seem to have been induced by the association procedure.

As in experiment 1, we assessed the possibility of a link between the reaction time measure that revealed a form of category learning and accuracy level; even if in the case of reaction times we did not expect any strong link with accuracy as these measures have been proposed to reveal different mechanisms (Prinzmetal et al., 2005). We conducted an analysis of the correlation between percentages of correct responses and the difference of reaction times between correct and incorrect responses during the post-learning phase. This analysis did not reveal any significant correlation between the two measures in either task (Pearson correlation: both *r* < 0.5, both *p*: ns).

Finally, post-experimental questionnaire revealed that in both tasks, 12 subjects out of 14 mentioned the eyes or eyebrows as particularly important for the task but only a minority of them reported having paid attention to the inter-eye distance (one in social task, three in non-social task). In order to test if the effects observed on reaction times were driven by the subjects who reported using inter-eye distance, we reproduced statistical analysis excluding these subjects. This did not change the overall pattern of results: RTs were shorter for correct than incorrect responses both in the social (correct responses: 840 ± 28 ms; incorrect responses: 866 ± 28 ms) and the non-social task [correct responses: 804 ± 38 ms; incorrect responses: 752 ± 55 ms; *F*(1,22) = 8.83, *p* < 0.05]. Thus it did not seem that the subjects who reported using inter-eye distance drove the effect observed on reaction times.

### Interim discussion

This experiment tested another association procedure: we used a passive exposure to the association between a category label and a physical feature. Although passive exposure did not induce a subsequent bias either in judgment accuracy or confidence ratings, it impacted categorization responses in the form of a slowing of reaction times for incorrect responses relative to correct responses in the post-association phase. These results are consistent with the findings of Barker and Andrade (2006) who also observed an influence of an experimentally induced association on reaction times in a social categorization task. The reaction time difference between correct and incorrect responses in post-association phase was observed in both social and non-social tasks. It reveals the existence of a form of knowledge of the association between category labels and physical feature.

Similar findings of reaction time modulation without accuracy improvement have been previously observed in the distinct field of visuo-spatial attention studies (Prinzmetal et al., 2005, 2010), even in situations where accuracy is at chance (Hsu et al., 2011). In line with these observations, the reaction time measure in our experiment was not found to be correlated to accuracy. Altogether these data suggest that reaction time reveal a different mechanism or, perhaps, a different stage of learning than the one revealed by accuracy measure. In particular it could reveal the learning of a form of unconscious knowledge. Indeed reaction time measure has been extensively used to reveal behavioral modifications when subjects are not explicitly aware of the nature of the experimental manipulation (e.g., Paller et al., 1987; Chaumon et al., 2008). This notwithstanding, the results of experiment 2 suggest that social categories are not systematically resistant to learning. Considering experiment 1 and 2 altogether further suggests some specificity of category learning, as we will now discuss.

## Discussion

This study aimed at examining the extent to which a short exposure (120 stimuli, ∼8 min) to a systematic association between a physical feature and a category label may impact performance differentially in two face categorization tasks that varied according to the nature of the category labels used (social versus non-social). Different association procedures were tested in two experiments: A feedback procedure was used in Experiment 1 while a passive exposure was used in Experiment 2. The results of these two experiments showed that non-social categorization was affected by the association between the physical feature and the category label induced by both the feedback and the passive exposure procedures. By contrast, social categorization was affected only when the association between the physical feature and the category label was enforced by passive exposure. This dissociation shows that social and non-social category acquisition are differentially sensitive to the association procedure. From this perspective, our findings support the view that social category learning is at least to some extent specific. They also emphasize the importance of the learning procedure used to study social category acquisition in the laboratory context.

Functional MRI studies have emphasized the existence of a dedicated neuroanatomical network involved in the *processing* of social categories (Zahn et al., 2007). We complement and extend these results by showing that social category *learning* is distinct from non-social category learning insofar as both feedback and passive exposure procedures impacted performance in our non-social categorization task whereas only the passive exposure procedure induced a significant impact on the performance in the social categorization task. These results are consistent with the hypothesis that learning mechanisms would be directly linked to the nature of categories (Ashby and Maddox, 2005; Cunningham and Zelazo, 2007). Indeed, our results underline the importance of the interaction between the learning procedure and the nature of the categories to be learnt.

First, our results underscore the importance of unsupervised learning and possibly group transmission in the acquisition of social knowledge. As both these unsupervised and group components of learning were jointly manipulated, it is unclear whether one of these components was more important than the other. Yet, it remains that the association procedure used in the second experiment seemed closer to the ecological conditions of person perception and social interactions, as compared to the reinforcement procedure through feedback, even if in a very controlled and simplified laboratory-based setting. Second, the kind of knowledge reinforced here was in the form of a statistical regularity in the association between a facial feature and a social category label. It is unclear to what extent social category learning may rely on the processing of such statistical regularity in the realm of social life. Indeed, whereas statistical regularities have been widely demonstrated to play a role in non-social visual category learning (Saffran et al., 1996; Fiser and Aslin, 2001; Sloutsky, 2003; Conway and Christiansen, 2006; Brady and Oliva, 2008) they have scarcely been studied in the social domain. Moreover, Tajfel (1982) proposed that social categorical judgments could be based on a large field of conceptual knowledge such as norms, values, stereotypes, attitudes. This notwithstanding, our results support the view that passive exposure to statistical regularities may also impact social categorization. This is in line with the results of Barker and Andrade (2006) who observed a modification of the nature of subject’s responses after they had been exposed to a statistical regularity in the form of an association between hair length and the traits of capability or kindness.

It is interesting to note that in the non-social task, feedback procedure, and passive exposure to the association influenced two distinct behavioral measures (confidence ratings and RTs respectively). This suggests a difference in the processing affected by the two association procedures.

Reaction time measures are largely used in implicit (or statistical) learning studies as a hint of the unconscious nature of subject’s knowledge (e.g., Paller et al., 1987; Chaumon et al., 2008; Vouloumanos, 2008; Pearce et al., 2010). Our findings of a RT effect in Experiment 2 when using a short passive exposure to an association may thus suggest that the knowledge acquired through a short passive is implicit in nature. The results of the *post hoc* questionnaires are in line with this hypothesis. As social categorization was affected only in the post-association phase of Experiment 2, this would fit with the view that social categorization processes take place implicitly for a large part (Uleman et al., 2005; Verosky and Todorov, 2010).

By contrast, confidence has been proposed as a meta-knowledge measure (as subjects are able to explicitly qualify the judgment they just made) and therefore could be an indication of the explicit nature of subjects’ knowledge (Dienes and Berry, 1997). It could therefore be tempting to speculate that the knowledge acquired through the feedback procedure would be more explicit in nature than the knowledge acquired through the passive exposure procedure. However, it is worth underlining that explicit knowledge is usually supposed to affect both accuracy and confidence judgments. In our experiment we did not reproduce this pattern but we nevertheless observed a correlation between accuracy levels and confidence ratings. However subjects who explicitly mentioned using inter-eye distance did not show larger effects on either confidence rating or accuracy level. We therefore do not have strong evidence that the association was explicitly acquired. Maybe the fact that our association phase was short (8 min, 120 trials) did not favor an emergence of behavioral effect on accuracy. Indeed, previous studies that observed learning evidences used longer learning phases: typically between 300 and 1000 trials (Nomura et al., 2007; Maddox et al., 2008; Spiering and Ashby, 2008; Li et al., 2009). This calls for caution in the interpretation of our confidence result as reflecting some explicit knowledge, and may rather suggest that confidence judgments constituted a more sensitive measure than categorization accuracy in our paradigm (Persaud et al., 2007).

Subtle learning effects were obtained as they concerned relatively indirect measures of learning while accuracy was not affected. Yet, these effects highlight the capacity for generalization of human category learning. Every face was seen only once in our experiments. Hence, whatever the knowledge acquired on the faces during the association phase, the fact that some indices of performance were modulated in the post-association phase indicates knowledge generalization to newly encountered faces. This generalization ability is reminiscent of previous finding obtained by Verosky and Todorov (2010) who showed that even a non-consciously perceived physical similarity between known and new faces can subtend a transfer of the knowledge (such as valence of the behavior) associated with the known faces for the evaluation of the new faces. Together with Verosky and Todorov (2010), our study shows that subjects are able to make inferences on the basis of a minimum amount of information shared between a known face and an unknown face. Both studies contribute to underscore the strong generalization ability of human learning on social stimuli such as faces.

Finally, it is interesting to note that our study used social categories of low-emotionality. This contrasts with previous studies (Barker and Andrade, 2006; Verosky and Todorov, 2010). In this respect, our results extend the knowledge about the human capacity to learn associations between physical features and social traits. They suggest that the perceptual salience of emotional stimuli is not strictly necessary to establish associations between physical and social traits, although it could facilitate their learning (Kleinsmith and Kaplan, 1963; LaBar and Phelps, 1998).

This study has several limitations. First, as mentioned above, the learning procedures of the first and the second experiments differed in two ways: a supervised (feedback) versus a passive learning procedures were used, and information was delivered in form of group judgments in Experiment 2. While taken as a whole, these differences allowed us to reveal a dissociation in social versus non-social category learning we cannot conclude about which of these two factors (feedback/passive learning and group information transmission) account for the observed differences. It will be interesting in futures studies to disentangle the role of passive learning and group information transmission in social category learning. Second, it may be noted that we contrasted category labels that are quite different in nature. Indeed, “flexible” and “determined” labels are semantically rich, they are related to complex social knowledge, whereas the “A” and “B” labels are abstract, meaningless labels, and in that respect seem to constitute less familiar labels. However, as proposed by Tajfel (1982), the complex knowledge content seems to be an intrinsic characteristic of social category labels. Furthermore, in everyday life it is not unusual to be separated in different arbitrary groups with “A” and “B” or “1” and “2” labels, such as at school or university when students are distributed into small study groups or classes. In this respect, the A/B non-social categorization task may not be considered as novel, and it had the advantage to be entirely devoid of social knowledge association. Nevertheless, it remains that the distinction between the “A” and “B” labels was likely to be less clear for the subjects than the distinction between “Flexible” and “Determined” labels: Subjects have more prior knowledge and probably also more consistent knowledge about the “Flexible/Determined” distinction than about the “A/B” distinction. This difference between our two types of categories constitutes a confounding factor that limits the generalizability of our results to social categories versus abstract non-social categories. Future studies will be needed to extend these results using semantically richer non-social categories. Finally, we would like to underline that the most important result of this study was related to the difference in the effects obtained with two different learning procedures (experiments 1 and 2) for the social task. This comparison highlighted that the learning of an association between social category labels and a physical feature of faces depends on the learning procedure.

In conclusion, our results highlight that social category learning may be highly dependent on the learning procedure and as such may bear some specificity as compared to non-social category learning. Our results show that social information is best acquired using an unsupervised association procedure mimicking a simplified form of group transmission, rather than by using feedback procedures. Last, our results underscore human flexibility and generalization ability when categorizing newly encountered faces. This remarkable ability might be a basis for the formation of inferences about unknown people that is essential to first impression formation.

## Conflict of Interest Statement

The authors declare that the research was conducted in the absence of any commercial or financial relationships that could be construed as a potential conflict of interest.

## Footnotes

^1^We checked that outlier exclusion did not change the overall pattern of results.
